# Parents’ perspectives of change in child physical activity & screen-viewing between Y1 (5-6) & Y4 (8-9) of primary school: implications for behaviour change

**DOI:** 10.1186/s12889-018-5445-2

**Published:** 2018-04-19

**Authors:** Russell Jago, Emma Solomon-Moore, Zoi Toumpakari, Deborah A. Lawlor, Janice L. Thompson, Simon J. Sebire

**Affiliations:** 1 Centre for Exercise, Nutrition & Health Sciences, School for Policy Studies, University of Bristol, 8 Priory Road, Bristol, BS8 1TZ UK; 2 Department for Health, University of Bath, Bath, BA2 7AY UK; 3 MRC Integrative Epidemiology Unit at the University of Bristol Oakfield House, Oakfield Grove, Bristol, BS8 2BN UK; 4 Population Health Sciences, Bristol Medical School, University of Bristol Oakfield House, Oakfield Grove, Bristol, BS8 2BN UK; 5 School of Sport, Exercise and Rehabilitation Sciences, University of Birmingham, Birmingham, B15 2TT UK

**Keywords:** Parents, Children, Screen-viewing, Physical activity, Qualitative, Interview

## Abstract

**Background:**

The aim of this study was to explore parents’ responses to changes in children’s physical activity and screen-time between Year 1 (5-6 years) and Year 4 (8-9 years of age) of primary school. A secondary aim was to identify how parents adapt their parenting to rapidly changing screen-based technology.

**Methods:**

Data were from the longitudinal B-Proact1v Study. Semi-structured telephone interviews were conducted between July and October 2016 with a sub-sample of 51 parents who participated in the study at Year 4. The sample was drawn from 1223 families who took part in the B-Proact1v in which the children wore an accelerometer for 5 days and mean minutes of moderate to vigorous intensity physical activity (MVPA) and sedentary minutes per day were derived. This sample was stratified according to the child’s MVPA and sedentary (SED) minutes per day, and by child gender. Data were thematically analysed.

**Results:**

Analysis yielded five main themes: 1) Parents reported how children’s interests change with free play decreasing and structured activity increasing. 2) Parents highlighted how their children’s independence and ability to make choices in relation to physical activity and screen-viewing increase, and that parental influence decreased, as the child gets older. 3) Parents reported that the transition from Year 1 to Year 4 appeared to be a time of substantial change in the screen-based devices that children used and the content that they viewed. 4) Parents reported that managing screen-viewing was harder compared to three years ago and a third of parents expressed concerns about the difficulty of managing screen-viewing in the future. 5) Parents reported using general principles for managing children’s screen-viewing including engaging the children with rule setting and encouraging self-regulation.

**Conclusions:**

Parents reported that children’s physical activity and sedentary screen behaviours change between Year 1 and Year 4 with children obtaining increased licence to influence the type, location and frequency with which they are active or sedentary. These changes and rapid advances in screen-viewing technology are a challenge for parents to negotiate and highlight a need to develop innovative and flexible strategies to help parents adapt to a rapidly changing environment.

## Background

Physical activity is associated with improved physical and psychological health and well-being among children [1]. There is also accumulating evidence that sedentary time, particularly sedentary screen-time, is associated with higher levels of cardio-metabolic risk factors [2] and adverse psychological well-being among youth [3]. There is, however, a debate within the field on whether these effects are related to, or independent of, physical activity [4]. As both physical activity and sedentary time track from childhood through to adulthood [5, 6], ensuring that children are as active as possible and minimising sedentary time are important for establishing the basis for an active lifestyle in later life. However, evidence suggests that children become less physically active and spend more time being sedentary as they age [7, 8]. For example, accelerometer data from the B-Proact1v study showed that girls mean counts per minute (CPM), an indicator of the volume of physical activity in which participants engage dropped from 686 CPM at Year 1 (5-6 years of age) to 587 CPM at Year 4 [8]. Thus, ameliorating these age-related changes is a key challenge.

While information on the age-related change in physical activity and sedentary time is critical for identifying the scale of the problem, this information provides few insights into how to change behaviours. Parents have been identified as key influences on children’s physical activity and screen-viewing [9–13], a ubiquitous sedentary behaviour amongst children and young people, both in terms of creating or limiting opportunities, and via parental attitudes which set the overall context for these behaviours in the household [10, 11]. An understanding of the factors that influence change, such as modifications in the child’s interest or adaptations in parents’ expectations, is important for identifying the types of strategies that parents can use to both promote physical activity and limit screen-time. This information could then be incorporated into behaviour change programs. A related challenge is to understand how both children’s and parents’ expectations of desired and acceptable screen-time change as children age.

Age-related changes in children’s physical activity and screen-time are occurring within a complex, constantly changing environment [14]. The increasing versatility and appeal of screen-viewing devices has the potential to increase sedentary time and limit the time and opportunities for physical activity. This change, which is likely due to rapid technological advances, mean that specific strategies to change behaviours related to a current form of technology will become redundant by the time their effectiveness has been evaluated. Despite previous assertions that physical activity and sedentary behaviour are distinct behaviours [15], the interplay between technology and sedentary time may suggest a need to consider related opportunities to increase physical activity and reduce sedentary time. This complexity is acknowledged in recent changes to the American Academy of Pediatrics children’s screen-viewing guidelines [16]. The new guidelines move away from setting time thresholds (e.g., two hours of screen-time per day) [17, 18] and instead recommend that: Parents and care-givers develop a family media plan that takes into account the health, education and entertainment needs of each child as well as the whole family [16]. As such, understanding parents’ responses to technological changes in the environment and technology are key to identifying potentially effective ways to manage screen-time and physical activity in a rapidly changing technological environment.

The aim of this study was to use in-depth qualitative methods to explore parents’ responses to changes in children’s physical activity and screen-time between Year 1 and Year 4 of primary school. A secondary aim was to identify how parents adapt their parenting around their child’s sedentary behaviour in the context of rapidly changing screen-based technology.

## Methods

Data are from the longitudinal B-Proact1v study, which aimed to examine factors associated with children’s and parents’ physical activity and screen-viewing behaviours. The study has been described in detail elsewhere [8, 19, 20]. Briefly, in 2012 and 2013, data were collected from 1299 Year 1 children (5-6 years old) from 57 primary schools across Bristol, UK. Between March 2015 and July 2016, 47 of the original schools were re-recruited and data were collected from 1223 Year 4 children (8-9 years old). At least one of the children’s parents were also recruited to the study. Children wore a waist-worn ActiGraph wGT3X-BT accelerometer during waking hours for five days including two weekend days. Accelerometer data were processed using Kinesoft (v3.3.75; Kinesoft, Saskatchewan, Canada), and were included in the primary analyses if children provided at least three days of valid data (including at least one weekend day). A valid day was defined as at least 500 min of data after excluding intervals of ≥60 min of zero counts, allowing up to two minutes of interruptions. Minutes spent in moderate-to-vigorous-intensity physical activity (MVPA) and mean sedentary time per day (SED) were derived using population-specific cut points for children [21].

Semi-structured telephone interviews were conducted between July and October 2016 with a sub-sample of 51 parents who participated in the study at Year 4. Telephone interviews were selected as the data collection method because they provide a cost-effective way of collecting information and allow flexibility for the participant and the researcher [22]. Only families with complete data for all measures (accelerometer and questionnaire data, child height, weight and blood pressure) were included in the potential interview sample (*N* = 625). This sample was stratified according to the child’s MVPA minutes per day (dichotomised around the study median: 57.5 min), sedentary minutes per day (dichotomised around the median: 434.6 min), and by child gender. This produced eight groups (1 = low MVPA, low SED boys; 8 = high MVPA, high SED girls). The order in which parents were invited to participate in an interview was randomised within each group. Participants were sent a £10 shopping voucher as reimbursement for participation in the interview. Interviewing continued until theoretical saturation was reached for the entire sample and the sub-groups. The study received ethical approval from the School for Policy Studies Ethics Committee at the University of Bristol, and written parent consent was received for all participants for parent and child participation [23].

### Interviews

An interview guide was developed and refined by the research team based on identifying gaps in current knowledge and further informed by the Year 1 B-Proact1v quantitative and qualitative findings [8, 19, 24–26]. Questions related to a variety of topics, including parents’ perceptions of their child’s physical activity and screen-viewing behaviours, strategies for managing these behaviours, understanding what has changed regarding these behaviours in the previous three years, and parents’ experiences from their own childhood. The interview guide, which has been previously published [27], included non-leading questions which allowed participants to shape the direction of the interview, and issues that emerged were probed. All interviews were conducted by two experienced members of staff aged 28-30 with previous interview experience. Interviews were conducted at the interviewee’s convenience (37 during weekday daytimes (72.5%), 13 during weekday evenings (25.5%), and 1 on a weekend evening (2%). The average interview duration was 35 min (range: 18 to 55 min). Of the interview participants, 31 were mothers and 20 were fathers, the average age was 41.2 (SD: 4.5) years, and 94.1% were White British.

### Data analysis

Interviews were transcribed verbatim and anonymised before being entered into QSR NVivo 10 (QSR International, Warrington UK) to facilitate analysis. The framework method [28] was used to inductively and deductively analysis the data [29, 30]. Hierarchies of categories were created and summarised, and illustrative quotes identified. The quantitative data were summarised to describe change in MVPA across the two time-points. Data on each participant including their gender, child’s gender and change in MVPA and SED is provided after each quote. For participants without change data, their interview group at Year 4 was provided.

## Results

A summary of the demographic profile of the parents and their children is presented in Table 1. Table 2 provides detailed information on the mean minutes of MVPA and SED at Year 1 and Year 4 for the 29 children with accelerometer data at both time points. As expected, based on the sampling frame, data indicate considerable variation in the profile of participants included in the study.

**Table 1:** Demographic characteristics of the interview sample of parents (*N* = 51) and their children

	Parents		Children	
	Mean (SD)	%	Mean (SD)	%
Gender (% female)		60.8		51.0
Age (years)	41.2 (4.5)	–	9.0 (0.4)	–
Body mass index (kg/m^2^)^a^	25.8 (6.1)	–	0.01 (0.95)	–
Index of multiple deprivation	11.5 (9.7)	–	–	–
Moderate-to-vigorous physical activity (mins/day)	48.1 (21.5)	–	58.3 (17.4)	–
Sedentary time (mins/day)	568.3 (149.3)	–	451.9 (103.6)	–
Ethnicity				
White British	–	94.1	–	–
Other	–	5.9	–	–
Employment				
Full-time	–	45.1	–	–
Part-time	–	39.2	–	–
Unemployed/full-time parent	–	15.7	–	–

^a^Body mass index value for children is BMI z-score based on the British 1990 Growth Reference

**Table 2:** Change in moderate-to-vigorous-intensity physical activity and sedentary time between Year 1 (5-6 years) and Year 4 (8-9 years) for children who provided data at both timepoints (*N* = 29)

Interview No.	Child gender	Moderate-to-vigorous physical activity (minutes per day)			Sedentary Time (minutes per day)		
		Year 1	Year 4	Change	Year 1	Year 4	Change
2	Boy	123.30	75.70	−47.60	390.67	537.37	146.70
4	Girl	73.61	72.39	−1.22	315.11	434.78	119.67
6	Boy	64.17	58.11	−6.06	334.67	493.83	159.16
7	Girl	49.25	64.07	14.82	280.29	749.80	469.51
10	Girl	35.83	16.33	−19.50	403.17	519.38	116.21
12	Girl	65.17	71.30	6.13	269.53	436.90	167.37
14	Girl	44.73	63.27	18.54	485.30	421.03	− 64.27
15	Girl	73.67	35.47	−38.20	375.07	476.93	101.86
16	Girl	51.33	57.17	5.84	395.46	426.00	30.54
17	Boy	47.93	68.67	20.74	334.23	423.30	89.07
18	Boy	68.27	85.83	17.56	402.57	364.13	−38.44
20	Boy	71.00	47.28	−23.72	334.50	420.39	85.89
23	Boy	54.97	63.17	8.20	404.10	390.46	−13.64
24	Girl	67.56	42.87	−24.69	372.56	430.67	58.11
25	Girl	53.67	83.87	30.20	328.00	351.33	23.33
27	Girl	56.75	54.61	−2.14	417.58	345.22	−72.36
29	Girl	62.83	56.25	−6.58	362.83	453.00	90.17
30	Girl	41.33	51.60	10.27	371.50	414.73	43.23
31	Girl	45.77	63.73	17.96	369.10	422.57	53.47
32	Boy	108.83	85.94	−22.89	243.50	413.28	169.78
33	Girl	33.33	48.53	15.20	468.33	456.17	−12.16
37	Boy	37.10	37.87	0.77	412.97	416.80	3.83
38	Boy	95.23	90.79	−4.44	314.27	373.79	59.52
40	Boy	52.80	33.60	−19.20	410.47	474.03	63.56
41	Boy	62.08	38.50	−23.58	358.71	315.13	−43.58
43	Boy	40.67	35.63	−5.04	374.67	527.30	152.63
45	Girl	63.75	51.25	−12.50	331.00	447.50	116.50
48	Boy	50.53	49.25	−1.28	442.77	482.96	40.19
51	Girl	70.13	75.70	5.57	305.53	421.93	116.40
Mean (SD) change:				−3.00 (18.71)			76.97 (102.86)

one participant (Interview 3) participated at Year 1 but did not provide valid PA data

The interviews and analysis yielded five main themes: 1) Change of child interests between Year 1 and 4; 2) Impact of child age on behaviour; 3) Change in the devices and content that are available to children; 4) Difficulties in managing screen-time; and 5) Principles of managing screen-time. Each of the five themes is presented in detail below.

### Change of interests between year 1 and 4

Parents referred to how children’s interests change, reporting that free play decreases with age, and that children move from free play to more structured activities, including those that they structure themselves.

*“when he does have down time, he does need something to kind of make him chill out…and playing with his toys or due to all that he’s not overly interested. He’s kind of getting older now and […] it’s not something that interests him so much.”* ***(Int 17, female parent, boy, MVPA increased, SED increased)***

*“I guess when he was younger he did a heck of a lot of imaginary play […], like sword fighting imaginary […] playing. Lego, statues, you know like figurines?[...] So he did a lot more of that when he was younger. So he would always be active but more like, like puzzles like Lego.”* ***(Int 23, female parent, boy, MVPA increased, SED decreased)***

*“And I think now a lot of their outside time, even when they’re on their own just our kids in the garden, they’re more, it’s more organised so they will, they have bike races with themselves, they’ll play football with themselves and they certainly do more football and hockey and swimming and stuff at school and organised things. So […] I guess the balance is moving towards more structured physical activity and away from freeform play.”* ***(Int 50, male parent, girl, Low MVPA, High SED)***

Parents also reported that their children’s interest in PA was maintained over time if they continued to enjoy and love PA but the form of physical activity changed becoming more structured and organised.

### Impact of child age on behaviour

Parents referred to their children’s independence and ability to make choices as they get older. Parents mentioned how children can play more independently in Year 4 compared to when they were younger, and how their physical activity may change when they are teenagers. Also, parents acknowledged that their influence on physical activity will decrease over the years.

*“… because she’s that bit older now she’s a bit freer to go around to her friends’ houses or to meet at the local park or what have you, that she couldn’t do three years ago cause she was younger, so, […] I – I think she’s – it’s just kind of a bit – a bit more independence for her really more than anything. I think that’s the – the major difference.”* ***(Int 4, female parent, girl, MVPA decreased, SED increased)***

*“Yes, he’s more active now […] than what he was when he was younger and I think that’s because he’s to – he’s older now to make choices...and now he’s a little bit older, he, he goes out and plays and he goes out on – because we live in a little cul-de-sac […], so he’s got a great environment to go out and play but he can go out and play and he’ll call on his friends by himself.”* ***(Int 32, female parent, boy, MVPA decreased, SED increased)***

It is, however, noticeable that only two parents referred to the impact of age on screen-viewing (Int 36, 39), with both referring to their child’s increased understanding of and independence to use screen-based technology without the parent’s supervision

*I: “And what about his screen viewing, do you feel that’s changed in the last few years?”*

*IV: “Er, it’s increasing as he gets older […], yeah. ‘Cause he’s more, more aware of the options I guess […] and is able to do it independently without help.”* ***(Int 36, female parent, boy, Low MVPA, Low SED)***

*I think it’ll become more of a challenge as they get older [mmm]. I think teenage girls and running around outside don’t necessarily go together [no] but yeah, I mean we’ll try and at the moment it’s fine.* ***(Int 50, male parent, girl, Low MVPA, High SED)***

*Um, I think, from what I see of friends’ children that are, that the sort of older they get they seem to kind of slow down, their, their physical activity unless their specifically involved in, in a specific sport which they’re either good at or really enjoy.* ***(Int 19, male parent, girl, Low MVPA, Low SED)***

*And I guess as he gets older, you know, we, as parents, it will be harder for us to influence him to do stuff, you know. [hmm] He'll have his own mind of what he wants to do* ***(Int 48, male parent, boy, MVPA decreased, SED increased)***

*At, at the ages they’re at, at the moment, yes [yeah]. I know as they sort of go – the girls go into sort of more teenage years, that’ll probably become more difficult. They won’t want to be with Mum and Dad. It won’t be so cool and [mmm] then it becomes more difficult, doesn’t it?* ***(Int 27, female parent, girl, MVPA decreased, SED decreased)***

### Change in the devices and content that are available to children

The transition from Year 1 to Year 4 appeared to be a time of substantial change in the devices that children used, how they used screen-based media what content they consumed. Parents commonly reported that in Year 4, their children owned a screen device (often a tablet or games console) that they did not own in Year 1, and some thought that this had increased their child’s screen-viewing independent of TV:

*“I bought her an iPad last year. Yeah, it’s…tripled. Well, it’s gone up nth-fold. Absol, she never had the iPad two years, from reception, so yeah, hugely increased without…not TV but, er, tablet, absolutely. […] Hugely increased.”* ***(Int 28, female parent, girl, High PA, Low SED)***

*“Erm, he, no different but I think probably in year one, I’m just thinking. Erm, they probably didn’t even have them devices in year one, I don’t think he had his Xbox then or the iPad so probably less device time – more device time now than in year one but he will have still had prob, probably more screen time and, and watching TV in year one than he does now.”* ***(Int 17, female parent, boy, MVPA increased, SED increased)***

Parents also reported changes in how their children engaged with screens compared to three years ago, with greater use of social media, online gaming, YouTube, on-demand media services (e.g., Netflix) and watching different shows on TV. It is useful to acknowledge that these new sources are not only providing new content but also new ways to efficiently view content without advertisements or watching other shows while waiting for the desired content to arrive. This could either be positive in terms of watching less or negative in terms of watching more via “binge” viewing. Parents commented on the rapid pace of change and a struggle to keep up:

*“The way that viewing has diversified with things like Netflix presenting itself as a, as a new opportunity for them to choose something, things like, the thing that’s really caught me off guard is the watching of the You Tube video […] which he wasn’t even aware of like a year ago and now Stampy is like, you know, a mini celebrity in the children’s world,… from watching, from watching um, Minecraft walk-throughs on, on You Tube.”* ***(Int 5, female parent, boy, High MVPA, High SED)***

*“I can’t think now if it was Year 1 or 2, erm, he had an iPad, but, erm, I think the online thing I think he didn't sort of get involved with until about Year 3 or 4. Erm, that’s – that’s when I would say things have more kind of changed when they get online and start talking to other – other friends from school and things, you know.”* ***(Int 18, male parent, boy, MVPA increased, SED decreased)***

Regarding the future, parents believed that screen-viewing content and particularly their child’s interest in it, and different aspects of it, will change again, directing children more into social media/online gaming. One parent described her feeling of inevitability that her daughter would become more interested in social media that she did not seem comfortable with her daughters’ future use of social media. There appeared to be a subtle sub-text of, and anxiety about, the future and that parents were seeking ways to navigate an increasingly complex online environment.

*“You know, and then obviously then she’s exposed to the whole world of, you know, all these apps and everything else that all these young girls want to go and post images of themselves. You know, she’s sheltered from that at the moment and, you know, not interested in it and, you know, none of the girls at school are talking about it but I know when she gets to secondary school they will.”* ***(Int 14, female parent, girl, MVPA increased, SED decreased)***

### Difficulties in managing screen-time

Parents reported that managing screen-viewing was harder compared to three years ago. Eighteen parents (35.3%) expressed concerns about the difficulty of managing screen-viewing in the future, due to children getting older and more independent, change in screen-viewing content, and an increased interest in screen-viewing and peer pressure from their friends.

*“When she was in Year 1, it would most definitely have been easier because she just really wouldn’t necessarily have wanted to go on anything at all [...] She might have wanted to watch a couple of CBeebies programmes and that would have been it.”* ***(Int 31, female parent, girl, MVPA increased, SED increased)***

Some parents felt that changes in their children’s use of mobile devices was a threat to their physical activity and interaction with other family members:

*“I think it’s getting worse, in regards to.... I’ve had it a couple of times with my daughter, she’s text me from upstairs and I’m downstairs, so I’m like, ‘No, if you want something you come and get it.’ So it’s even that type of little – I know it’s not a lot, but – not physical activity but just walking up and down and interaction with people.”* ***(Int 6, male parent, boy, MVPA decreased, SED increased)***

Parents also felt that their ability to guide their children’s screen-viewing would reduce as their children grew older and as the content becomes more engaging:

*“they’ve not turned into teenagers yet but it, I don’t know how long they’ll respect me saying, ‘No, you can’t turn the telly on’.”* ***(Int 39, female parent, boy, Low MVPA, Low SED)***

*“I think they do – his generation now, as I said, probably do spend too much time on these, er, you know, iPads and Xboxes and things like that really, but, er, it’s just difficult to sort of try and get them off, I guess. It’s, er – like I said, it is quite a social thing because they’re all talking to each other, so it’s, erm, yeah.”* ***(Int 18, male parent, boy, MVPA increased, SED decreased)***

Some parents were concerned that they did not fully understand the screen-viewing technology themselves:

*“I feel like I should know more than they do and I don’t know that I do anymore! [Laughs] But probably myself. […] Erm, to some extent I think it’s quite sweet that they’ll call me a klutz, I don’t know what I’m doing, but on the other hand it worries me that they can manipulate things faster than, than I can.”* ***(Int 28, female parent, girl, High PA, Low SED)***

Some parents reported feeling conflicted about restricting screen-time because of the educational, social, and relaxing benefits of some forms of screen-viewing.

*“I think watching television can be, can be good, I like television myself and always have done I think. It can be a good medium, umm educational and entertaining, so, so I’m not umm, so I’m not evangelical against TV.” (* ***Int 44, male parent, boy, High PA, Low SED)***

The parents also clearly indicated that they had not thought about how things would change as the child ages or how their parenting approach would need to adapt.

*“ difficult to answer really, I don’t think I’ve made any particular plans and I’ll address the issue should they arise.”* ***(Int 2, female parent, boy, MVPA decreased, SED increased)***

“*no, not structured plans, no [okay]. Manage as it, as it evolves [yeah]”.* ***(Int 46, male parent, girl, High MVPA, High SED)***

*“I hadn't thought that far ahead yet.”* ***(Int 47, male parent, girl, Low MVPA, Low SED)***

### Principles of managing SV

Parents reported using some general principles for managing children’s screen-viewing. These included engaging the children with rule setting, and encouraging self-regulation:

*“I think it’s important to engage the children in it because they can also self, you know, regulate it as well in a way, […]if you want to be on that PlayStation with your friend for four hours here that’s fine but that means you don’t do it here. So engaging them in you know what is appropriate and what isn’t probably is a good idea.”* ***(Int 2, female parent, boy, MVPA decreased, SED increased)***

*“Yeah, they have to be part of the, the deal there […], I think. There has to be some compromise as well because you – they’ll respond better if, if they – you’ve listened to them as well and the compromise, so yeah […]. Definitely, making the rules together is, is good.”* ***(Int 30, female parent, girl, MVPA increased, SED increased)***

Some parents commented on the importance of setting a good example by role-modelling desired screen-viewing behaviours, but thought this would be challenging given their own use of screens for work, social media and communications:

*“I think most parents are pretty hypocritical about erm, about their screen viewing. So the parents will happily tell the kid to stop looking at a tablet while they sit there merrily, you know, writing texts, or emails, or playing on Facebook or whatever.”* ***(Int 45, male parent, girl, MVPA decreased, SED increased)***

*“I think that we are going to have to become more adept as parents at setting good examples for our children by having rules for ourselves that we can then, because as they become older it’s going to be much harder for us to be um, you know, having a rule for us where we are just on our phones whenever we want and then expecting them to, to limit their time.”* ***(Int 5, female parent, boy, High PA, Low SED)***

*“Erm, er, so I think, I, I think, I think consistency is really important […] and I mean certainly, I mean... my, my wife spends a lot of time sat on her tablet writing emails and trying to, trying to do – trying to get her admin done erm, so – but the problem is, of course, the, the children see her doing it.”* ***(Int 45, male parent, girl, MVPA decreased, SED increased)***

## Discussion

The data presented in this paper have shown that parents perceive that their children’s physical activity and sedentary screen-time behaviours exhibit marked changes between the beginning (Year 1) and middle (Year 4) of primary school. They report particularly notable changes with respect to increases in the time spent using game consoles to play online with friends and watching videos on YouTube. This finding is consistent with the objective data from this project which showed that accelerometer assessed mean minutes of MVPA decreased by 3 min per day for boys and 7 min for girls between Year 1 and Year 4 while mean sedentary time increased by 20% for boys and 23% for girls [8]. The paper therefore provides qualitative reinforcement of the key quantitative findings. The data also provided evidence that parents recognise that the technological environment is always changing and the necessary constant adaptations that are required by them is a challenge, especially as many parents expressed that they often struggled to keep up with changes in technology themselves. Findings, therefore, highlight a need to increase support for parents to manage their children’s physical activity and screen-viewing, and that this support needs to take account of age and rapid technological changes.

Age has differential impacts on physical activity and sedentary screen-time. As children age, the licence that that their parents provide for them to make their own decisions about when and how they are active or sedentary increases. Previous research has also shown that children’s independent mobility to be physically active changes as they move from primary to secondary school [31], and reinforces the need to identify ways to embrace the increased licence as an opportunity for increased physical activity and limiting sedentary time. Similarly, as the options and desire for screen-viewing increase, limiting screen-time becomes more of a challenge. There is also the paradox where parents are happy to give children increased independence when they have a mobile phone [32] with which they can be contacted. As such, they are simultaneously giving them more freedom to be active while providing them with a portable screen-viewing device and encouraging them to use it.

The findings in this paper suggest that parents need to constantly adapt the approach they take to the management of their children’s physical activity and screen-time to take account of changes in preferences, technology hardware and the technological environment (i.e., different and emerging platforms) in which the child engages. Moreover, as parental control over child behaviour weakens as children age [10], the parent-child interactions will also need to adapt to make greater use of less direct control and greater use of negotiations and collaborative goal setting. It is also important to highlight that many parents reported they expect the management of their children screen-viewing to become more difficult as the children get older, but as they felt the issue was currently manageable most did not have plans on how to manage physical activity and screen-viewing as the children age. This suggests a need to help parents plan for issues that are likely to arise as their children move through primary school.

Previous work has shown that although consistent messages within families are important for managing youth screen-time [33, 34], the content of the message may need to constantly evolve. This may suggest that less specific guidelines about how to manage screen-time and promote physical activity that are not so reliant on individual behaviours would be helpful. For example, negotiating rules with children about when, where, with whom and how often screen-time could be engaged in could be advocated, regardless of whether the behaviour in question is watching cartoons or online game playing [16]. Furthermore, encouraging parents to model the screen-viewing behaviours that they wish their children to adopt may be helpful for establishing the overall structure of the conversations around limits. Families could be encouraged to develop and agree on their shared view on physical activity (e.g., its importance, how much they do, and how to support each other), irrespective of whether this is playing catch in the garden or encouraging walking to school without parent support. These discussions would also need to take account of the broader environment which differs in terms of safety, accessibility, equipment availability and parental willingness to afford greater licence. Thus, although the offer and specific forms of support and management provided to children may change as they age, the way in which parent support is provided which is often termed as the parenting style [35] and the underlying way of conveying this support could be consistent.

Table 3 summaries the key principles for managing child physical activity and sedentary time that have emerged from this study. These key principles are flexible and could form part of discussions between parents and their children and encourage the development of shared solutions as opposed to a source of conflict. Furthermore, as managing physical activity and screen-viewing is expected to become more difficult as the children age, the middle of primary school appears to be a good age to develop parent and child self-regulation skills that will be useful later on in adolescence.

**Table 3:** Key findings and implications for behaviour change programs

Finding	Implication for behaviour change programmes
Physical activity interests change as children age, moving from free-play to structured activities	Identify times in day to promote physical activity and flex the content to match changes in interest
Parental influence on PA and SV becomes less overt – more about facilitation, support and modelling	Need to develop parental facilitation skills and encourage parents to model the behaviours that they wish their child to adopt
Children want increased licence for both physical activity and sedentary time as they age	Provide children with a range of nearby PA options to encourage participation with friends and independent mobility without parent support – explore this in next year’s interviews? Develop ways to encourage children to use increased licence to engage in active options as opposed to sedentary screen options
Devices and technology constantly evolve	Develop a shared family view on screen-viewing that is focussed on the time / setting and not the device
Child knows more about screen-viewing devices than parent	Encourage child to share knowledge with parent to build shared understanding of the technology and how to use it
Screen-viewing interests change	Develop key principles for screen-viewing that can adapt as interests change

*PA* Physical Activity

*SV* Screen-viewing

### Strengths and limitations

One of the major strengths of this study is the embedding of qualitative research into a larger cohort study. This process facilitated the recruitment of participants based on their objectively-measured levels of physical activity with a good level of variation in socio-economic position, and with a sample that includes 20 fathers which is approximately 40% of the sample. This sampling process has enabled us to explore the experiences of a variety families as their children’s behaviour and the technological environment has changed. The result is a very rich and unique dataset that has provided novel insights into an important public health area. Moreover, the robustness of the data collection and analysis process has provided a rigorous evaluation of the area, and there was clear saturation of information in the analyses. The study is however limited as it was only conducted in one large city in the Southwest of England, and as such the ability to extrapolate to other settings and countries is limited.

## Conclusion

Parents feel that their children’s physical activity and sedentary screen behaviours change between school Years 1 and 4 with children obtaining increased licence to influence the type, location and frequency with which they are active or sedentary. These changes are a challenge for parents to negotiate. They expressed concern about the rapid changes in screen-viewing technology, which was seen as posing a particular challenge for parents. These findings highlight a need to develop innovative, flexible strategies to help parents adapt to a rapidly changing environment. Parents need help both to manage the change between Year 1 and Year 4, and for the future.

## Abbreviations

CPM: Counts Per Minute
Int: Interview
MVPA: Moderate to Vigorous Intensity Physical Activity
SD: Standard Deviation
SED: Sedentary minutes per day
